# Timing of sexual debut and associated sociodemographic and HIV risk factors among young people in Eswatini

**DOI:** 10.1371/journal.pone.0303942

**Published:** 2024-06-14

**Authors:** Garikayi B. Chemhaka, Maswati S. Simelane

**Affiliations:** Department of Statistics and Demography, University of Eswatini, Kwaluseni, Manzini, Eswatini; Human Sciences Research Council, SOUTH AFRICA

## Abstract

Young people in sub-Saharan Africa and specifically in Eswatini (previously Swaziland), continue to be disproportionately affected by HIV despite having equitable access to antiretroviral treatment. Early sexual debut is one of the many factors linked to HIV infection that is discussed in the context of general public health. Monitoring this behavior is essential for developing preventative, evidence-based interventions. This study aims to describe the early and/or typical sexual debut among young people in Eswatini and examines sociodemographic and HIV risk factors associated with early and typical sexual debut timing. We analyzed cross-sectional secondary data from the 2016/17 Swaziland HIV Incidence Measurement Survey (SHIMS), which had a representative sample of 2,383 young people aged 18–24. Respondents were selected using a two-stage stratified probability sampling design. We applied descriptive statistics and multivariable multinomial logistic regressions to examine the data. Out of the 2,383 respondents, 71.3% had sexual experience, with 4.1% and 26.5% reporting early sexual debut (<15 years) and typical sexual debut (<18 years), respectively. Our study found that age, sex, education, marital status, wealth, sexual partners in the past 12 months, and alcohol use were significantly associated with early and/or typical sexual debut. It is crucial to consider the sociodemographic factors and HIV risk factors of young people when designing programs and interventions aimed at preventing early sexual debut or transition. This approach is necessary to promote better sexual and reproductive health in alignment with sustainable development goals.

## Introduction

Age at first sexual debut is a crucial life course indicator of sexual risk as it indicates the beginning of exposure to HIV infection [1, 2] and represents the sexuality of young people [3]. Age at which individuals engage in their first sexual intercourse varies across different regions and countries [4–6] with definitions ranging from before age 15 [4, 7–9] to the age 18 [5, 10–12]. For instance, in Tanzania, 14.3% of young males and 9.1% of females reported having had sex before the age of 15 [13]. In South Africa, overall, 13% of youth (7.6% females versus 19.5% males) reported having had sexual intercourse before age 15 [14].

In Eswatini, a country with a generalized HIV epidemic, 3.4% of young people had sexual intercourse before the age of 15, that is, 4.5% males versus 2.5% females [15].Early sexual debut in Eswatini is associated with a higher incidence of HIV infection among young people aged 15–24 years. In 2017, among young people, 9.1% were HIV positive, 4.1% males versus 13.9% females [15]. The burden of HIV was higher among young people aged 20–24 years (4.2% males and 20.9% females) compared to those aged 15–19 years (3.9% males and 7.2% females) [15]. In Eswatini, young people frequently experience obstacles obtaining modern protective contraceptives including condoms, leaving them susceptible to HIV infection [16, 17].

Early sexual debut is associated with subsequent risky sexual behavior such as multiple concurrent sexual partners, and inconsistent condom use, which increase the risk of HIV infection [1, 5, 18, 19], and poor sexual and reproductive health outcomes as was found in Ghana [6], Malawi [20], South Africa [19], and Nepal [21]. Other unintended consequences include child marriage, and childhood or unwanted pregnancy [6, 22]. Delaying sexual debut is therefore an important strategy to improve the health and well-being of young people as reflected in the sustainable development goals (SDGs), specifically goals number 3 to 5 [23]. Therefore, understanding the factors associated with early sexual debut is crucial to designing targeted interventions meant to delay sexual debut among young people in Eswatini.

Studies have identified sociodemographic and HIV-related factors that are associated with early sexual debut. These factors include age, sex, marital status, household wealth index, number of sexual partners, condom use, HIV testing, and location of residence [7, 19, 20, 24–26]. These factors can be classified as protective or risk factors, which either decrease or increase the risk of early sexual debut [27].

The problem behavior theoretical perspective suggests that early sexual debut is considered a behavior that deviates from social expectations for adolescents [27–29]. It is associated with norm-breaking, and a desire to seek adulthood status and privileges [29]. Hence, adolescents would engage in delinquency, alcohol abuse, substance abuse, and multiple cohabiting or sexual relationships [27, 29, 30]. The stage termination hypothesis suggests that young people who experience early puberty may be more likely to engage in sexual activity at younger ages than those who mature later, especially for females [29]. This hypothesis proposes that the timing of puberty is related to the age at sexual debut, with early-maturing individuals potentially facing difficulties navigating the developmental transitions that occur early in life due to their limited readiness and abilities [28, 31]. Adolescents may struggle with negotiating for safe sex, and anticipating and emotionally preparing for sexual activity due to their limited dating experiences or simply because they feel ashamed about using condoms [1, 21].

The patriarchal cultural contexts, as highlighted by socialization argument, influence the timing of sexual debut among young people. Socialization perspective highlights that men often dominate sexual activity and condom use in such contexts [18, 20]. In addition to cultural roles and gender dynamics, socioeconomic status is associated with the timing of sexual debut in a manner that lower socioeconomic status is often associated with a higher likelihood of early adolescent sexual initiation [6, 22, 29]. Young people of lower socioeconomic status often have fewer options or opportunities in life and may become more susceptible to premarital or early sex [29].

It is therefore necessary to better our understanding of the various sociodemographic and behavioral factors associated with early sexual activity among young people to make pragmatic sexual and reproductive policies or interventions in the context of sub-Saharan African countries [27]. Since societal differences in the timing of sexual debut are little understood in Eswatini, it is critical to identify groups that are more likely to engage in risky sexual activities. Consequently, by comparing early sexual timing experiences to the late or delayed debut group, this study aimed to close this gap [30, 31]. Our study aims to determine sociodemographic and HIV risk (and protective) factors of early sexual behavior (early and typical sexual debut) among young people aged 18–24 years in Eswatini, a country with a generalized HIV epidemic. The first objective of this study is to describe the timing of sexual debut, including age thresholds for early, typical, and late debut, among adolescents and young people in Eswatini. The second objective is to assess the association between sociodemographic and HIV risk factors, and the timing of sexual debut among 18-24-year-olds in Eswatini.

## Materials and methods

### Study setting, design, and participants

The study was conducted in Eswatini, one of the smallest southern African countries with a high HIV epidemic estimated at 27% [15]. A two-stage stratified probability sampling design was used for the data collection. In the first stage, enumeration areas (EAs) which became the primary sampling units (PSUs) were identified and selected by using the 2007 census of Swaziland. The PSUs were stratified by urban-rural and region status. In the second stage, a sample of households was collected using systematic random sampling from the selected EAs. All adults aged 15 years and above in the sampled households were interviewed [15].

The study participants were selected from a cross-sectional national representative population survey of the 2016/17 Swaziland HIV Incidence Measurement Survey (SHIMS). Trained field enumerators conducted face-to-face interviews using tablets to collect sociodemographic and behavioral information. The study sample consisted of young adults aged 18 to 24 years. Participants below 18 years were excluded as were not expected to have a late sexual debut, resulting in a subset final sample of 2,383 sexually experienced young individuals.

### Ethical approval

The study analyzed secondary data from the 2016/17 Swaziland HIV Incidence Measurement Survey (SHIMS), which was obtained from the Population-based HIV Impact Assessment (PHIA) project protocol of Columbia University. The study protocol received ethical approval from the Swaziland Scientific and Ethics Committee [15]. Informed consent was obtained before the interview and participants were guaranteed confidentiality and anonymity of their responses as indicated in the SHIMS report [15]. The SHIMS data analyzed in this study were anonymous. The data are publicly available upon request on their website: https://phia.icap.columbia.edu.

### Measures

The dependent variable is the timing of sexual debut, which was obtained by asking respondents their age at first sexual intercourse, regardless of gender or circumstances. Specifically, participants were asked ‘how old were you when you had sex for the first time?’ In alignment with recent legislation in Eswatini that defines a child as someone under 18 years [32], and based on the literature [9, 28, 31], we classified the timing of sexual debut as ‘early’, ‘typical’, and ‘late’ for those who had their first sexual encounter at less than 15, 15–17, and 18 years and older, respectively. We then coded the respective reported ages as 0, 1, and 2 (reference category).

The explanatory variables of interest in this study include age (adolescents (18–19 years) versus young adults (20–24 years), gender (female versus male), ever married (yes versus no), education (primary education or less, secondary school or high school and tertiary), area of residence (urban versus rural), and household wealth (poor, middle, rich). Household wealth was measured in quintiles (poorest (1) to richest (5)) using principal component analysis of household materials and goods. We classified the two first quintiles as poor and the upper two quintiles as rich.

The study also examined key HIV risk (or protective) factors, such as sexual partners in the past 12 months (none, one, multiple (2 or more)), condom use at first sex (yes versus no), condom use at last sex (yes versus no), ever tested for HIV (yes versus no), and alcohol consumption in the last 12 months (none, moderate and hazardous/misuse). To measure alcohol consumption, we used the identification test for consumption (AUDIT-C) scale instrument developed by Saunders et al. [33], which categorizes the possible 12-point score for alcohol use as none (score 0), moderate (scores 1–2), and misuse/hazardous drinking (scores 3–12) [34]. The SHIMS questionnaire’s three-dimensional standardized 4-point Likert items on alcohol use disorders on ‘frequency’, ‘quantity’, and ‘binge’ alcohol use are the basis for the classification. The AUDIT-C scale has high validity [34] and has been applied in prior studies for Eswatini [35, 36].

### Data analysis

Data were analyzed using Stata version 14. The SHIMS predefined complex survey weights were applied using a Jackknife replication technique to adjust for under coverage, nonresponses, and variable probability selection of the sample [15]. Missing data were excluded from the analysis. Univariable analysis was done to show the frequencies and percentages of all variables. Bivariable analyses were conducted using the Chi-square test to examine the relationship between the dependent variable and all explanatory variables. Finally, multinomial logistic regressions with robust standard errors were conducted using the three-level outcome; timing of sexual debut (early, typical, and late). Late debut was set as the base/reference, and two models were used. First, in unadjusted regression, all explanatory variables were independently regressed onto the outcome. Second, all explanatory variables were entered simultaneously into a multivariable regression to identify parsimonious predictors using a stepwise approach, irrespective of the statistical significance found in bivariable analyses. Associations were described using relative risk ratios (RRR) along with 95% confidence intervals (95% CI). Significantly associated variables were determined using an alpha (α) level of 0.05 (P<0.05).

## Results

### Characteristics of respondents

Of the 2,383 young people aged 18–24 years included in this study, the majority (70.2%) were aged 20–24 years and female (51.6%). The mean age was 20.92±2.01 years. Most of the participants had never been married (88.4%), had ever tested for HIV (83.2%), never consumed alcohol (79.1%), completed secondary or high school education (74.5%), and resided in a rural area (75.2%). In terms of wealth status, 55% of the respondents were from non-poor households, with 24.5% belonging to the middle wealth status and 31.1% to the rich/. Nearly two-thirds of respondents had either one or multiple sexual partners in the past year. Almost half of the young people (51.4%) did not use condoms at their first sexual debut while slightly more than half of the respondents (56.4%) did not use condoms at last sexual encounter (Table 1).

**Table 1:** Sample description and bivariable analysis of selected variables by the timing of sexual debut in Eswatini, 2016/17.

	Timing of Sexual Debut					
Characteristic	Early (%)	Typical (%)	Late (%)	Total (%)	N	P-value
**Age (mean ± sd)**	21.17±1.87	20.80±2.01	20.95±2.02	20.92±2.01		<0.001*
18–19	20.6	32.4	29.4	29.8	711	
20–24	79.4	67.6	70.6	70.2	1672	
**Gender**						<0.001*
Female	39.7	58.7	49.7	51.6	1231	
Male	60.3	41.3	50.3	48.4	1152	
**Ever married**						0.304
No	79.8	81.3	91.6	88.4	2106	
Yes	20.2	18.7	8.4	11.6	277	
**Education**						<0.001*
Primary/less	26.5	23.0	15.0	17.6	419	
Secondary/high school	64.5	71.4	76.2	74.5	1774	
Tertiary	9.0	5.6	8.8	7.9	189	
**Household wealth**						0.173
Poor	35.2	46.3	44.2	44.4	1058	
Middle	25.8	25.7	23.9	24.5	584	
Rich	39.0	28.0	31.8	31.1	742	
**Area of residence**						0.101
Urban	36.2	23.8	24.5	24.8	592	
Rural	63.8	76.2	75.5	75.2	1791	
**Region of residence**						0.164
Hhohho	30.7	30.9	29.5	29.9	713	
Lubombo	17.3	18.9	18.6	18.6	444	
Manzini	36.2	30.0	35.2	33.8	806	
Shiselweni	15.7	20.1	16.8	17.6	420	
**Sexual partners in past 12 months**						0.006
None	9.4	7.9	46.6	34.8	829	
One	65.3	70.2	44.5	52.2	1243	
Multiple	25.3	21.8	8.9	13	311	
**Condom use at first sex**						0.018
No	42.9	33.9	58.6	51.4	1224	
Yes	57.1	66.1	41.4	48.6	1159	
**Condom use at last sex**						0.156
No	43.0	41.3	63.0	56.4	1345	
Yes	57.0	58.7	37.0	43.6	1038	
**Ever tested for HIV**						0.080
No	12.3	11.5	19.1	16.8	400	
Yes	87.7	88.5	80.9	83.2	1983	
**Alcohol consumption**						<0.001*
None	54.4	70.6	83.9	79.1	1886	
Moderate	17.0	14.2	8.8	10.6	252	
Misuse	28.6	15.3	7.3	10.3	245	
**Type of sexual intercourse**						
None	0	0	41.5	28.8	685	
Anal	3.0	1.3	1.2	1.3	31	
Vaginal	97	98.7	57.3	70.0	1667	
**Total**	98	632	1652	100	2383	
Sexual debut timing **(mean ± sd)**	13.20±1.14	16.28±0.78	19.14±1.26^	17.73±2.08~		
Sexual debut timing (Total %)	4.1	26.5	69.3	100		

*p<0.05, sd = standard deviation, N = number of observations, ^N = 932, ~N = 1671

Of the total respondents, 71.3% had sexual experience (70% for vaginal sex versus 1.3% for anal sex). Slightly above two-thirds (69.3%) reported a late sexual debut (at 18 years or later) compared with 4.1% who had an early debut (first sex before 15 years) and 26.5% who had a typical debut (first sex at age 15 and before 18 years). Young people had an early, typical, and late sexual debut at an average age of 13.20±1.14, 16.28±0.78, and 19.14±1.26 years, respectively. The overall mean age of sexual debut was 17.73±2.08.

The Chi-square results showed the timing of sexual debut was associated with age, gender, education, number of sexual partners in the past 12 months, condom use at first sex, and alcohol consumption. Having completed secondary or higher education, not having sexual partners in the past 12 months, not using condoms at first sex, and not drinking alcohol were associated with a late sexual debut. Alternatively, primary or less education, one or multiple sexual partners, condom use at first sex, and moderate to hazardous alcohol use were risk factors for an early or a typical debut.

Chi-square tests were used to compare individuals who had an early and typical debut. Among those who had an early debut, 60% were males and 39.7% were females. On the other hand, females (58.7%) were more likely to have had a typical debut compared to males (41.3%) counterparts. Most young people (79.4%) who reported having had an early sexual debut were aged 20–24 years. The results indicated no significant association between the timing of sexual debut and having ever been married, household wealth, condom use at last sex, ever been tested for HIV, living in urban/rural residence, or region of residence (see Table 1).

Overall, the study found that respondents who reported being aged 20–24 years, never married, having completed secondary or high school education, having one sexual partner in the past 12 months, using a condom at first and/or last sex, having ever tested for HIV, not using alcohol, living in rural areas and residing in the Manzini region were more likely to have an early or typical sexual debut (Table 1).

### Characteristics associated with early and typical sexual debut

The study conducted multinomial logistic regression models for unadjusted and adjusted timing of sexual debut in Eswatini, which are shown in Table 2. In the unadjusted multinomial analyses, age, gender, education, area of residence, sexual number of partners, condom use at first sex, condom use at last sex, being ever married, and alcohol consumption were associated with the timing of sexual debut. Early sexual debut was positively associated with age and residing in an urban area while a typical sexual debut was positively associated with females and being ever tested for HIV. Both early and late sexual debut were positively associated with being ever married, primary or less education, number of sexual partners, condom use at first sex, condom use at last sex, and level of alcohol consumption. The timing of sexual debut was not associated with household wealth. Additional information on the risk factors for early and typical sexual debut based on the unadjusted relative risk ratio (URRR) is presented in Table 2. The significance and directional influence of explanatory variables on the timing of sexual debut was similar in both unadjusted and adjusted multinomial regression, except for age. Therefore, the significant results in the adjusted parsimonious multivariable model are interpreted in more detail for brevity.

**Table 2:** Unadjusted and adjusted multinomial regression model showing correlates of timing of sexual debut in Eswatini, 2016/17 (N = 2,383).

	Unadjusted		Adjusted	
Late (base category)	Early	Typical	Early	Typical
Characteristic	URRR (95% CI)	URRR (95% CI)	ARRR (95% CI)	ARRR (95% CI)
**Age**				
18–19 (Ref.)	1	1	1	1
20–24	1.61 (1.01–2.55)*	0.87 (0.69–1.09)	0.61 (0.34–1.09)	0.36 (0.26–0.48)***
**Gender**				
Female (Ref.)	1	1	1	1
Male	1.5 (0.95–2.36)	0.69 (0.56–0.86)***	1.44 (0.87–2.39)	0.76 (0.59–0.98)*
**Ever married**				
No (Ref.)	1	1	1	1
Yes	2.76 (1.63–4.69)***	2.51 (1.88–3.34)***	2.22 (1.16–4.23)*	1.55 (1.1–2.17)*
**Education**				
Primary/less (Ref.)	1	1	1	1
Secondary/high school	0.48 (0.3–0.75)**	0.61 (0.49–0.77)***	0.49 (0.29–0.83)**	0.65 (0.5–0.86)**
Tertiary	0.58 (0.23–1.49)	0.41 (0.25–0.7)**	0.38 (0.14–1)*	0.41 (0.22–0.76)**
**Household wealth**				
Poor (Ref.)	1	1	1	1
Middle	1.35 (0.71–2.58)	1.03 (0.78–1.36)	1.56 (0.8–3.04)	1.15 (0.87–1.52)
Rich	1.54 (0.93–2.54)	0.84 (0.64–1.1)	1.72 (1.02–2.89)*	0.95 (0.68–1.31)
**Area of residence**				
Urban (Ref.)	1	1		
Rural	0.57 (0.35–0.95)*	1.04 (0.8–1.35)		
**Region of residence**				
Hhohho	1	1		
Lubombo	0.89 (0.47–1.68)	0.97 (0.72–1.3)		
Manzini	0.99 (0.53–1.83)	0.81 (0.61–1.09)		
Shiselweni	0.9 (0.4–2)	1.14 (0.84–1.56)		
**Sexual partners in past 12 months**				
None (Ref.)	1	1	1	1
One	7.26 (3.27–16.12)***	9.26 (6.61–12.97)***	6.8 (2.8–16.54)***	11.42 (7.56–17.25)***
Multiple	14.04 (6.05–32.63)***	14.33 (9.51–21.61)***	9.93 (3.65–26.99)***	18.2 (11.22–29.53)***
**Condom use at first sex**				
No (Ref.)	1	1		
Yes	1.88 (1.18–3)**	2.75 (2.21–3.42)***		
**Condom use at last sex**				
No (Ref.)	1	1		
Yes	2.26 (1.54–3.3)***	2.42 (1.99–2.95)***		
**Ever tested for HIV**				
No (Ref.)	1	1		
Yes	1.68 (0.93–3.03)	1.81 (1.31–2.5)***		
**Alcohol consumption**				
None (Ref.)	1	1	1	1
Moderate	2.98 (1.56–5.71)**	1.91 (1.38–2.64)***	2.16 (1.11–4.19)*	1.62 (1.15–2.29)**
Misuse	6.06 (3.5–10.5)***	2.49 (1.8–3.44)***	3.88 (2.02–7.46)***	2.16 (1.43–3.26)***

*p<0.05

**p<0.01

***p<0.001, Ref. = reference category

In the adjusted multivariable analysis, the results in Table 2 are generally the same as those of the unadjusted analyses when independent variables are simultaneously controlled for. Overall, the findings indicate a significantly higher probability of early and typical sexual debut for those who have ever been married, are primary or less educated, had one or more sexual partners, and drank alcohol moderately or hazardously. Age was negatively associated with both early and typical sexual debut, but not significant for the former. The study found that young adults aged 20–24 were less likely to have had a typical debut (adjusted relative risk ratio [ARRR] = 0.36, 95% CI: 0.26–0.48) compared to those who had a late debut, using age adolescents (18–19 years) as the reference group. The odds of typical, but not early, debut relative to late debut were lower for males (ARRR = 0.76, 95% CI: 0.59–0.98) than females. Among young people who had early and typical debut compared to those who delayed debut, the ever-married individuals were more likely to have had an early and a typical debut (ARRR = 2.22, 95% CI: 1.16–4.23 and ARRR = 1.55, 95% CI: 1.10–2.17, respectively) than their never-married peers.

In the study, it was found that young people with secondary or high school education (ARRR = 0.49, 95% CI: 0.29–0.83) and tertiary education (ARRR = 0.38, 95% CI: 0.14–1.00) were less likely to have an early sexual debut than those with primary or less education. Similarly, those with secondary or high school education, and tertiary education were 0.47 times and 0.36 times, respectively less likely to have a typical debut than those with primary or less education. Relative to late debut, young people from rich households were 1.72 times more likely to have an early debut compared to those from poor households (ARRR = 1.72, 95% CI: 1.72–2.89).

When young people with an early debut were compared to those who had a late debut, those who had one sexual partner (ARRR = 6.80, 95% CI: 2.80–16.54) and multiple sexual partners (ARRR = 9.93, 95% CI: 3.65–26.99) had a higher likelihood of having an early debut than those who did not have a sexual partner. The odds of both early debut and typical debut were strongest among young people with one or multiple sexual partners. Young people who had one sexual partner (ARRR = 11.42, 95% CI: 7.56–17.25) or multiple sexual partners (ARRR = 18.20, 95% CI: 11.22–29.53) in the past 12 months were 11.4 times and 18.6 times more likely to have had a typical debut than late debut compared to those who had no sexual partners. Moderate drinkers (ARRR = 2.16, 95% CI: 1.11–4.19) or hazardous drinkers (ARRR = 3.88, 95% CI: 2.02–7.46) were 2.2 and 3.9 times more likely to debut early than late compared to young people who are non-alcohol drinkers. Similarly, the odds of a typical debut increased with both moderate and hazardous drinking (ARRR = 1.62, 95% CI: 1.15–2.29 and ARRR = 2.16, 95% CI: 1.43–3.26). Both early and typical debutants had considerably greater proportions of young individuals reporting hazardous or moderate alcohol usage compared to late debutants. Those who had an early debut had the strongest associations with alcohol misuse.

## Discussion

The primary objective of this study was to contribute to the existing body of literature on the timing of sexual debut among young people, which is presumed an understudied area. The specific aims were to describe the timing of sexual debut and examine its associations with sociodemographic and behavioral HIV risk factors among young people in Eswatini. The findings indicated that 71.3% of young people had engaged in sexual activity at some point. Slightly more than two-thirds (69.3%) of young people in this nationally representative sample had delayed or postponed their sexual debut. Among those who had an early sexual initiation, 4.1% reported having an early debut (<15 years) while 26.5% reported a typical debut (15–17 years). The prevalence of early sexual debut is relatively low compared to a previous study for sub-Saharan African countries with a similar study sample of 18-24-year-olds which showed a prevalence ranging from 8.6% to 17.7% [9].

Consistent with previous research [18], which suggests that sexual debut typically occurs between the ages 15 and 18 years in sub-Saharan African countries, our study found that the mean age of sexual debut was 17.73±2.08 years, with a mean age of 13.20±1.14 for early debut and 16.28±0.78 for typical debut. Those who had a late debut had a mean age of 19.14±1.26. The mean age of early debut was similar to that reported in other studies conducted in Africa [16, 18, 20, 26, 37].

Using data from a population-based cross-sectional survey conducted in Eswatini, this study employed multivariable models to assess the timing of sexual debut and its associated HIV risk (or protective) factors among young people. The findings revealed that the timing of sexual debut was associated with several sociodemographic factors, including age, gender, being ever married, household wealth, and education, as well as HIV-risk factors such as sexual partners in the past 12 months, and alcohol consumption. For brevity, early (<15 years) and typical (<18 years) sexual debut timing in the literature [8–11] can both be used to refer to the initiation of early sexual activity or behavior.

In this study, we found that young adults aged 20–24 years had lower odds of a typical sexual debut compared to their adolescent peers aged 18–19. This finding is consistent with previous research conducted in Malawi [20], Ghana [6], and some East African countries [25]. Adolescents are vulnerable to early sexual behavior due to their lack of sexual experience, peer pressure, and societal constructions of gender roles that encourage taking sexual risks [1, 6]. The results reveal that, in comparison to those who had a later debut, males were less likely to be associated with a typical debut than females. Previous studies have reported earlier sexual initiation among males than females in diverse settings and posit that males are more adventurous [38], experience less parental monitoring [27], and face fewer social sanctions [6, 31] than females. An early debut is often socially endorsed for men to prove their sexual prowess while proscribed for women who are deemed to be minors and have more to lose from sexual activity [6, 27]. On another note, young women could be afraid of social consequences when they disclose their early sexual exposure [38, 39]. However, in some contexts as in this study, the opposite has been reported for females having sexual debut much earlier than males [20, 21]. Our results suggest that young sexually active people in Eswatini are more likely to contract HIV if they are female and adolescents.

Compared to late debut, young people who had been ever married had a higher risk of both early and typical debut, as found in other studies [4, 25]. Although sexual intercourse is morally acceptable in marriage in many societies [18], early sexual activity is noted to be exceptionally higher among young people who cohabit [30]. Early sexual initiation is often frowned upon in most cultures, particularly among adolescents and young people [39]. In Eswatini, premarital sex is discouraged by religious and traditional fraternities that promote chastity among boys and girls through laws and customary practices [40]. However, teenage pregnancy and childbearing are common among women in Eswatini, as it proves their reproductive potential in the marriage market [17]. Young women may secure marriage quite early and may have little or no control over their sexual activity due to various layers of authority over them in a patriarchal society [20]. Women are often sanctioned in gender power relations, which shapes their first and later sexual activities and makes many of their sexual experiences risky for HIV infection and other consequences [21].

The literature highlights the protective effect of formal education against early sexual debut [10, 18, 25, 27]. Our study found that an early and typical sexual debut were both inversely associated with the level of education. Specifically, young people with an early debut or a typical debut were less likely to have secondary or higher educational outcomes compared to those with a late debut. This aligns with previous studies that found a negative correlation between the timing of sexual debut and the level of completed education [6, 18, 31]. Early sexual debut is associated with poor educational outcomes. Adolescents and young adults who had primary or less education are relatively more likely to debut early since they are less likely to advance their academic careers. Young people with better education tend to delay their sexual debut than those with basic or no formal education, as this helps them focus on their future goals and avoid adverse consequences [6, 21]. Eswatini introduced sex education or literacy in the high school curriculum in 2014 [40]. This finding further supports the hypothesis that education is protective against HIV infection [19, 27].

Our study found that the number of sexual partners in the past 12 months was positively associated with both early and typical sexual debuts among young people. This variable appeared to have had the highest and strongest influence on early sexual behavior when compared to other characteristics. Young people who had an early or typical debut were more likely to have had multiple sexual partners in the past 12 months than their counterparts who had no sexual partner, compared to those who had a late debut. This finding supports previous studies that have found an association between having multiple sex partners and age at first sex [1, 38]. Early sexual debut is assumed to have a relationship with meeting several sexual partners over time [21, 30]. A possible explanation for this relationship could be prescribed cultural norms and mores that reinforce manhood by having multiple sexual partners while women are considered minors with little or no control over their bodies [20].

Contrary to expectation, an early sexual debut was positively associated with household wealth. However, there was no significant association between household wealth and a typical sexual debut. Specifically, young people from rich households were more likely to debut early than their counterparts from poor households. This finding contradicts previous studies that have found lower socioeconomic status (wealth) associated with reporting earlier initiation of sexual intercourse [22, 29]. Household wealth may work in reverse ways for men and women on their experience of first sexual activity. While wealth may be a motivator for males to engage in sexual activity, poverty is the factor that drives women to engage in sexual activity [18]. Community engagement, by involving care givers, community members, healthcare providers, and partnership organizations, can create a supportive environment, and foster positive social norms for young people including those from rich households.

We found that alcohol consumption was positively associated with the timing of sexual debut. Specifically, young people who had an early or typical debut were more likely to have had moderate or hazardous alcohol use than those who had a late debut. This finding is consistent with previous studies from Nigeria [38], Ethiopia [10, 26], and other resource-poor and high-income countries [7, 37] which found an association between alcohol consumption and early sexual exposure. Alcohol abuse may result in a lack of self-control that could affect the onset of early sexual activity [38]. Our study supports a previous study by Durowade et al. [38], which found that alcohol consumption had the strongest influence on early debut.

Our findings suggest that alcohol misuse increases the risk of HIV transmission among young people in Eswatini. This is consistent with a study conducted in Eswatini, which reported that 10.9% of people living with HIV had a history of alcohol abuse [35]. Alcohol consumption is socially accepted in several cultural events and yearly festivities in Eswatini [35, 40], it has been linked to HIV stigma in some settings, which may discourage individuals from seeking HIV treatment and prevention services [1, 41].

The results indicate that both early and typical debutants face similar outcomes, including reporting lower/primarily or less education, moderate or hazardous alcohol use, being adolescents (age 15–19), being ever married, and being male. However, the significance of being male was only observed for an early debut and not for typical debut.

Preventions programs and policies that are backed with evidence and address the specific needs of young people in Eswatini must continue to be provided to facilitate necessary skills that delay sexual intercourse. Given the study findings, there is tremendous need for comprehensive educational initiatives among in-school and out-of-school youths that focus on behavioral changes, particularly in relation on gender equality or norms, alcohol consumption, condom use and sexual partnerships. Therefore, educational programs should provide accurate information, develop decision making skills and promote safe sexual behavior. Behavior change interventions should empower young people, and encourage responsible sexual behavior. Awareness should be raised about the risks of alcohol misuse and rehabilitation for individuals struggling with alcohol-related issues should be provided. Educational opportunities for young people should be strengthened to reduce the likelihood of engaging in early sexual activity due to limited options. Addressing the early transition to sexual intercourse among young people should be done before they initiate sexual intercourse to understand their sexual needs [27].

Our study has several limitations. Although the study is based on a cross-sectional nationally representative survey, the responses are self-reported and subject to errors due to underreporting, recall bias, and social desirability bias. When obtained, certain cross-sectional data, such as marital status, condom use during last intercourse, and place of residence, may not always be indicative of the time when the exposure (sexual debut) occurred. Additionally, the question of first sex did not differentiate between vaginal and anal intercourse which may be important in sexual behavior studies. Comparing unequal age bands 18–19 and 20–24 for adolescents and young adults, respectively may be problematic although it is of theoretical relevance.

The majority of young people in Eswatini who reported transitioning to first sexual intercourse under the age of 18 had a typical debut (age 15–17 years) rather than an early debut (age less than 15 years). The study highlights the influence of sociodemographic factors such as age, gender, marital status, wealth status, and level of education, as well as HIV risk factors such as sexual partners, and alcohol consumption, on the timing of early and typical debut. The study emphasize the importance of considering sexual debut as a multi-category response rather than a binary outcome, and suggests future research should utilize longitudinal designs to assess changes in sociodemographic and behavioral characteristics.

In conclusion, this study provides valuable insights into the complex dynamics of early and typical debut among young people in Eswatini. The findings underscore the need for comprehensive and targeted interventions that address the sociodemographic and behavioral factors influencing early sexual activity. By promoting education, behavioral changes, and supportive policies, it is possible to empower young people to delay their first sexual intercourse and engage in safer sexual practices, ultimately improving their health and well-being in alignment with sustainable development goal number 3. It is important to note that some early sexual debut is likely to persist despite such interventions and thus the feasibility and practicality of such interventions should be considered by policy makers. Thus, measures should be considered alongside other things such as encouraging availability, awareness and use of primary HIV prevention methods given the study findings indicated an association between early sexual debut and sexual behaviors known to be associated with increased risk of HIV infection. Strengthening education, interventions, and legislation is crucial to reduce or prevent early sexual activity and its associated risks.
